# Anti-Oxidant, Anti-Inflammatory and Anti-Angiogenic Properties of Resveratrol in Ocular Diseases

**DOI:** 10.3390/molecules21030304

**Published:** 2016-03-02

**Authors:** Allan Lançon, Raffaele Frazzi, Norbert Latruffe

**Affiliations:** 1 Laboratory BioperoxIL, Faculty of Sciences SVTE, University of Bourgogne, 6 Bd Gabriel, F-21000 Dijon, France; allan.lancon@gmail.com; 2 Laboratory of Translational Research, Arcispedale S. Maria Nuova IRCCS, Reggio Emilia 42123, Italy; raffaele.frazzi@asmn.re.it

**Keywords:** resveratrol, eyes, inflammation

## Abstract

Resveratrol (3,4′,5 trihydroxy-trans-stilbene) is one of the best known phytophenols with pleiotropic properties. It is a phytoalexin produced by vine and it leads to the stimulation of natural plant defenses but also exhibits many beneficial effects in animals and humans by acting on a wide range of organs and tissues. These include the prevention of cardiovascular diseases, anti-cancer potential, neuroprotective effects, homeostasia maintenance, aging delay and a decrease in inflammation. Age-related macular degeneration (AMD) is one of the main causes of deterioration of vision in adults in developed countries This review deals with resveratrol and ophthalmology by focusing on the antioxidant, anti-inflammatory, and anti-angiogenic effects of this molecule. The literature reports that resveratrol is able to act on various cell types of the eye by increasing the level of natural antioxidant enzymatic and molecular defenses. Resveratrol anti-inflammatory effects are due to its capacity to limit the expression of pro-inflammatory factors, such as interleukins and prostaglandins, and also to decrease the chemo-attraction and recruitment of immune cells to the inflammatory site. In addition to this, resveratrol was shown to possess anti-VEGF effects and to inhibit the proliferation and migration of vascular endothelial cells. Resveratrol has the potential to be used in a range of human ocular diseases and conditions, based on animal models and *in vitro* experiments.

## 1. Introduction

Resveratrol or 3,4’,5 trihydroxy-trans-stilbene is a natural polyphenol. It was first identified in 1940 in the root of the white hellebore [1]. Later, resveratrol was also found in large quantities in the root of Japanese knotweed *(Polygonum cuspidatum)* [2]. In 1976, Langcake and Pryce discovered resveratrol in vine, as produced by the grape plant in response to biotic infections or abiotic stresses, such as UV irradiation or exposure to ozone [3]. Resveratrol is produced by a wide variety of plants, some of which are edible by humans. It is also found in trees, such as the butterfly tree or pine, in legumes, such as rhubarb, in peanuts and also in a number of berries: grapes, blackberries, blackcurrants, blueberries, and cranberries. Nevertheless, it is in grape, and, more precisely, in grape skin, that the concentration of resveratrol is at its highest. Thus, red wine is the most concentrated food source of resveratrol found in the diet of man.

Resveratrol is one of the best-studied phytophenols with pleiotropic properties. In addition to being a phytoalexin in vine, leading to a stimulation of the natural defenses of the plant, resveratrol exhibit many beneficial effects in animals and humans, by acting on a wide range of organs and tissues. These include the prevention of cardiovascular diseases, anti-cancer potential, neuroprotective effects, homeostasia maintenance, aging delay, and a decrease in inflammation.

Breakthroughs were as follows: (1) In the prevention of cardiovascular disease, resveratrol limits platelet adhesion, promotes coronary vasorelaxation, and even reduces arrhythmia [4]; (2) as an anti-cancer molecule, resveratrol could sensitize cancerous tumors to chemotherapy [5]; (3) resveratrol counteracts certain neurodegenerative diseases, such as Alzheimer’s disease, where it appears that neuronal cell death, induced by β-amyloid peptides, is also limited by resveratrol [6]; (4) resveratrol modulates metabolism by improving insulin sensitivity in humans [7]; (5) resveratrol contributes to elongating lifespans [8]. Upon an induced high-calorie diet, induced obese mice used to die younger, but regained a normal lifespan by consuming resveratrol [9]. Finally, (6) resveratrol exerts anti-inflammatory properties.

The inflammation is the result of chemokine/cytokine secretion (also called interleukins). Chemokines are small chemotactic proteins, which mobilize leukocytes by making them migrate from blood circulation towards the place of inflammation. These chemokines bind to their receptors at the membrane of monocytes to activate them to macrophages in order to eliminate pathogens or damaged cells and tissues. One possibility to reduce inflammation is to inhibit the production of chemokines. The inflammation process is generally associated with pain, and some studies have reported the analgesic [10] and anti-inflammatory properties of resveratrol [11]. Moreover, it has been recently shown that resveratrol inhibits the expression of pro-inflammatory genes in human monocytes by stimulating the synthesis of anti-inflammatory microRNAs [12]. The synthesis of pro-inflammatory lipid mediators from arachidonic acid can occur via several pathways, including the prostaglandin H synthase (PHS) pathway, the cyclooxygenase (COX) pathway, and the lipoxygenase (LOX) pathway. Most anti-inflammatory drugs target the production of inflammatory mediators from arachidonic metabolism through cyclooxygenases 1 and 2 (COX-1 and -2), which are involved in the synthesis of pro-inflammatory leukotriens and prostaglandin E2 (PGE2) [13,14].

This paper reports the literature concerning the effects of resveratrol on the phenomena involved in ocular diseases, by focusing on the anti-oxidant, anti-inflammatory, and anti-angiogenic properties of this molecule. For instance, age-related macular degeneration (AMD) is one of the main causes of deterioration of vision in adults in developed countries.

## 2. Results and Discussion

### 2.1. Antioxidant Effects of Resveratrol

Although the exact pathogenesis of AMD is not completely clear, most studies indicate an early role of the retinal pigment epithelium (RPE) in the onset of the disease, with smoking as the most important environmental risk factor. RPE cells are involved in phagocytosis and the degradation of photoreceptor outer segments, a phenomenon that necessitates large amounts of energy and oxygen. As a result, the tissue undergoes a continuously high level of oxidative attacks that stress the epithelial cells and can lead to their death by apoptosis. This early stage of AMD development is most likely favored by the ingestion of cigarette smoke [15]. Indeed, the products of tobacco combustion contain a high concentration of free radicals and toxic compounds, such as benzopyrene or acrolein, which increase oxidative damage.

Due to its recognized antioxidant activity, resveratrol has been used in several studies to determine its ability to protect cells of the RPE (retinal pigment epithelium) from oxidation and, thus, estimate the potential value of this molecule as a preventive or curative treatment for AMD. In 2010, Sheu *et al.* [16] analyzed the toxic effects of acrolein, a powerful oxidant molecule. They reported that resveratrol showed beneficial effects at relatively low concentration (10 μM) on the RPE cells, including the lifting of the inhibition of phagocytosis, induced by acrolein, and protection against the toxicity of the acrolein/H_2_O_2_ cocktail.

Furthermore, Pintea *et al.* [17], working on RPE cells, reported the protective effects of polyphenols against cytotoxicity induced by hydrogen peroxide. Used in pretreatment, resveratrol was able to induce a significant and dose-dependent increase of enzymatic activities of the antioxidant defense system: superoxide dismutase, glutathione peroxidase, and catalase. In addition, resveratrol was able, under both normal and oxidative stress conditions, to increase the level of reduced glutathione, an antioxidant molecule naturally synthesized by the cells. Finally, resveratrol inhibited the production of reactive oxygen species (ROS) by RPE cells, a finding which supports the hypothesis that this compound may also participate in antioxidant defenses by capturing free radicals directly *in cellulo* (a phenomenon also known as scavenging).

Similar effects were observed in lens cells. This organ is particularly sensitive to oxidative damage because the fiber cells that constitute it are not renewable and have a limited lifespan. The accumulation of damage to these cells throughout the life of an individual causes the degradation of proteins and eventually leads to cataracts.

In a study performed on human lens epithelial cells, Zheng *et al.* [18] showed that resveratrol reduces cell death, as well as the accumulation of ROS, following oxidative attack by H_2_O_2_. This cell protection appears to be mediated by the increased expression of defense enzymes, such as superoxide dismutase-1 (SOD-1), catalase, and heme oxygenase-1 (HO-1).

Glaucoma is another pathology that can be caused, in part, by oxidative attack. Although inflammation seems to be the main trigger of the disease (see the following section), oxidative stress is a contributing factor by altering the operation of the trabecular meshwork. This alteration leads to a lack of circulation of aqueous humor and finally to ocular hypertension.

In a study focusing on markers of glaucoma, Luna *et al.* [19] showed that resveratrol provides a protective effect by normalizing the production of ROS in cells of the trabecular mesh when they were subjected to oxidative stress by hyperoxygenation.

Resveratrol, thus, increases the level of natural antioxidant defenses in various cell types of the eye.

It also seems to increase the *in vitro* activity of defense enzymes and the level of intracellular antioxidant molecules to limit the formation of free radicals (ROS) and the onset of irreversible damage, and initiators of diseases, such as AMD, cataracts, or glaucoma.

### 2.2. Anti-Inflammatory Effect of Resveratrol

**Figure 1:**
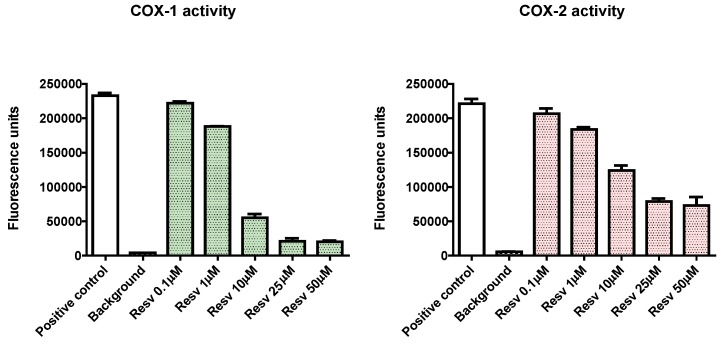
Inhibitory effects of Resveratrol on COX1 and COX2 activities, two enzymes involved in the synthesis of pro-inflammatory leukotrienes. Resv, resveratrol; COX-1 and COX-2, cyclo-oxygenases 1 and 2 [27].

Inflammation is a phenomenon that is often associated with oxidation. It has been implicated in the onset of diseases such as arthritis, psoriasis, Crohn’s Disease, cardiovascular diseases, and cancer. Interestingly, numerous polyphenols (epigallocatechin-gallate, curcumin, genistein) inhibit COX genes expression [20]. Moreover, resveratrol inhibits COX-1 and COX-2 activities in a dose-dependent manner (Figure 1) [21]. This result is consistent with resveratrol binding into the active site of COX-1, thus impairing COX-1 binding to arachidonic acid and, therefore, inhibiting its catalysis. In addition, the direct binding of resveratrol to COX-2 is required to inhibit cancer cell growth [22]. Resveratrol down-regulates COX-2 expression by acting on NF-κB and the AP-1 complex transcription factors, which are under the control of the signaling kinases: IκκBα processing to IκBα p50/p65 and MAPK/ERK/p38/JNK. In this study, resveratrol prevents the phosphorylation of IκκBα and MAPK [23]. These findings support some potential therapeutic indications based on natural polyphenols (such as resveratrol) aimed at substituting, at least in part, steroidal or non-steroidal anti inflammatory drugs (abbreviated as SAID or NSAID respectively) with the aim to alleviate pain.

Some years ago, it was widely recognized that inflammatory processes were also is playing a key role in the mechanisms of retinal diseases, such as diabetic retinopathy [24] and AMD [25].

Kubota *et al.* [26] have performed an *in vivo* study of ocular inflammation. Uveitis was induced in a mouse model by endotoxins (EIU) and the protective effects of resveratrol were assessed. This study showed that five days of prevention by oral supplementation with resveratrol was able to inhibit the production of two proteins crucial in the course of the inflammatory process: Inter Cellular Adhesion Molecule-1 (ICAM-1) and Monocyte Chemoattractant Protein 1 (MCP-1). The MCP-1 protein is a chemokine expressed by endothelial cells lining the vasculature. Its role is to attract immune cells, such as leukocytes to the inflammatory site. These leukocytes are then hooked by ICAM-1 proteins present on the surface of the endothelium, to extract and distribute them from the bloodstream to the target tissue.

Resveratrol anti-inflammatory effect is thus exerted by reducing the adhesion of leukocytes to the wall of retinal vessels.

In diabetic retinopathy, the persistence of high levels of blood glucose causes chronic inflammation with a slow but progressive deterioration of RPE cells leading to the impairment of the blood-retinal barrier and the loss of central vision. Losso *et al.* [28] used retinal pigment cells to study the inflammatory phenomenon triggered by a condition of hyperglycemia. They showed that cells subjected to this stress secrete pro-inflammatory cytokines, such as interleukin 6 and interleukin 8, and that resveratrol inhibits their production in a dose-dependent manner. At the same time, the activity of COX-2 (responsible for the production of pro-inflammatory prostaglandins) is also inhibited by resveratrol, whereas the protein expression of cellular interaction connexin 43 and Gap-junction are increased. As a consequence, cellular cohesion is maintained, thus preventing the degradation of the blood-retina barrier.

Luna *et al.* [19] observed that resveratrol protective effect comes from its inhibition of the production of ROS in cells of the trabecular meshwork that were subjected to oxidative stress by hyperoxygenation, a factor that can promote the initiation of glaucoma. They revealed that resveratrol also has the ability to decrease the expression of inflammation markers, such as interleukin-1α, interleukin-6, interleukin-8 and E-selectin. The latter, also known as ELAM-1 for Endothelial Leukocyte Adhesion Molecule-1, is involved in the same way as ICAM-1 in recruiting leukocytes to the site of inflammation.

**Figure 2:**
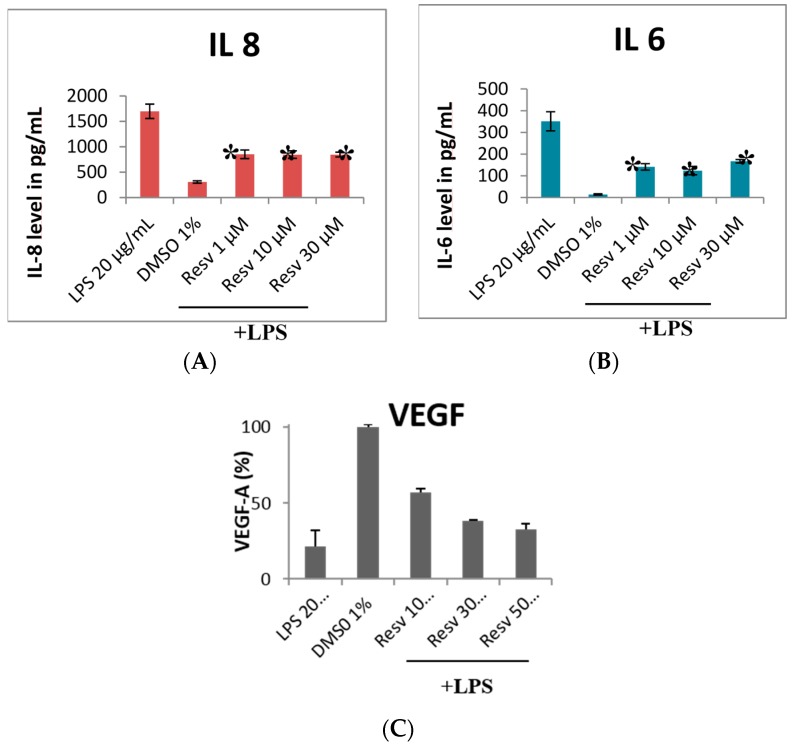
Interleukins and VEGF-A assays on resveratrol-treated retina ARPE-19 cell lines. Data are mean ± SD (*n* = 3); 1% of solvent DM SO was used as a vehicle in all treatments. Mann-Whitney Test * *p* ≤ 0.05.

Using human retinal pigment epithelial cells (ARPE-19 cell line), our laboratory has also shown that resveratrol has anti-inflammatory potential (see Supplementary Materials). These effects arise from the decrease of IL-6 and IL-8 interleukins expression (Figure 2A,B) [29]. In addition, due to its anti-oxidant properties, resveratrol exhibits a cytoprotective effects against the enhancement of IL-8, MCP-1, and VEGF secretion in ARPE-19 [20]. Based on these studies, it can be concluded that resveratrol play preventive effects on the pathogenesis of wet AMD by decreasing the associated low grade inflammation, thus decreasing IL-6 secretion, and consequently maintaining a low level of neutrophil chemo-attraction [30].

### 2.3. Anti-Angiogenic Effect of Resveratrol

Angiogenesis is a normal and necessary process in the development of the organism and in tissue repair. This process becomes abnormal when, for example, it is diverted to further the growth, such as in AMD.

Brakenhielm *et al.* [31] reported the effects of resveratrol in a model of corneal neovascularization in mice. The induction of angiogenesis was performed by implanting corneal tissue with wicks soaked in growth factors. The effect of vascular endothelial growth factor (VEGF) and fibroblast growth factor type 2 (FGF-2) resulted in the appearance of a dense neovascularization around the periphery of the cornea. Consumption of resveratrol in drinking water for three days before implantation of the wicks limited the area of neovascularization induced by VEGF and FGF-2 and the density of the network as well.

Losso *et al.* [27] study of RPE submitted to hyperglycemia-induced stress confirmed this inhibitory activity on VEGF secretion.

Hua *et al.* [32], using another model of pathological development of neovascular lesions *in vivo*, evaluated the therapeutic effects of resveratrol. Unlike the corneal neovascularization model presented above, these researchers used mice mutants for the receptor of Very Low-Density Lipoprotein (VLDLR). Unexpectedly, after 15 days of treatment with resveratrol, these mutant VLDLR^−/−^ mice spontaneously developed retinal neovascularization and photoreceptor degeneration, similar to those encountered in macular telangiectasia and retinal angiomatous proliferation in man. The study showed that consumption of resveratrol in mice, starting five days before the first appearance of neovascularization, decreased the development of all lesions observed on the entire retina at 30 days by 70%.

In addition, in its preventive effect, resveratrol also shows therapeutic potential. Indeed, a supplementation in resveratrol beginning six days after initial injury was still able to slow the progression of neovascularization by approximately 40% at 60 days.

Using retinal pigment epithelial ARPE-19 cell line, our laboratory has shown that resveratrol exerts anti-angiogenic potentials (see Supplementary Materials). These effects are accomplished by the inhibition of VEGF-A secretion (Figure 2C) [27]. Again, the anti-angiogenic effects of resveratrol seems to be mediated by its anti-VEGF action. This hypothesis was validated by the normalization of the VEGF mRNA level by resveratrol in VLDLR^−/−^ mouse retina at 60 days [32].

Resveratrol anti-VEGF property *in vivo* may be associated with anti-proliferative and anti-migrative effects on metastasis, as demonstrated by the authors in an *in vitro* experiment on human osteosarcoma cells [33].

The anti-angiogenic effect of resveratrol demonstrated *in vivo* and *in vitro* by this recent research has also been demonstrated in cancer in terms of anti-metastatic activity. Indeed, beyond its anti-VEGF, anti-proliferative, and anti-migrative effects on vascular endothelial cells, resveratrol also has the ability to inhibit the degradation of the extracellular matrix by metalloproteinases, thus reducing the development of new vessels [34,35].

All together, the above results suggests that resveratrol may be considered as a promising therapeutic agent in eye diseases associated with neo-angiogenesis, such as AMD, macular telangiectasia, and retinal angiomatous proliferation.

In perspective, eye pathologies could also benefit from the pleiotropic effects of resveratrol. This compound specifically acts in the context of oxidative and inflammatory stress, often linked with neovascular complications.

Many papers have established [36] that resveratrol is able to act on various cell types of the eye by increasing the level of natural antioxidant enzymatic and molecular defenses. Resveratrol anti-inflammatory effects are due to its capacity to limit the expression of pro- inflammatory factors, such as interleukins and prostaglandins, and also to decrease the chemo-attraction and recruitment of immune cells to the inflammatory site. Finally, resveratrol was shown to possess anti-VEGF effects and to inhibit proliferation and migration on vascular endothelial cells. These three effects collectively explain the resveratrol anti-angiogenic action.

Although all these discoveries have been made *in vitro* or *in vivo* in mice, resveratrol use in humans should require complementary studies. A strategy for the treatment or prevention of angiogenic eye diseases by means of a resveratrol-based treatment could be particularly interesting because it involves three fronts: anti-oxidation, anti-inflammation, and anti-angiogenesis. Resveratrol has the potential to be used in a range of human ocular diseases and conditions, based on animal models and *in vitro* experiments. Thus far, it is not currently used in the clinic or for animals. Further studies are thus needed.

## Supplementary Materials

Supplementary materials can be accessed at: http://www.mdpi.com/1420-3049/21/3/304/s1.
