# Supplementary material for: Anti-Oxidant, Anti-Inflammatory and Anti-Angiogenic Properties of Resveratrol in Ocular Diseases
Source: Molecules. 2016 Mar 2;21(3):304. doi: 10.3390/molecules21030304 (PMC6272926; doi:10.3390/molecules21030304)
Supplement: Supplementary file 1 [file molecules-21-00304-s001.pdf]

Supplementary Materials: Anti-Oxidant, Anti-Inflammatory and Anti-Angiogenic Properties of Resveratrol in Ocular Diseases

Allan Lançon, Raffaele Frazzi and Norbert Latruffe

1. Measurement of COX-1 and COX-2 Activity

Trans-resveratrol (Resv) was acquired from Sigma-Aldrich (St. Louis, MO, USA). It was tested using the COX fluorescent inhibitor screening assay kit (Cayman, Ann Arbor, MI, USA) according to the authors of Reference [1]. Scalar concentrations (specified in the graphs) of Resv were tested on purified, ovine COX-1 and human recombinant COX-2. The commercial inhibitors SC-560 and DuP-697 active on COX-1 and COX-2 isozymes, respectively, were used as controls.

The fluorescent product of the reaction (Resorufin; excitation wavelength 530–540 nm; emission wavelength 585–595 nm) was quantified using a fluorimeter (Fluostar Omega, BMG labtech, Ortemberg, Germany).

2. Estimation of *in Vitro* Anti-Inflammatory and Anti-Angiogenic Properties

Cultured human ARPE-19 cells (human retinal pigment epithelial cells) (American Type Culture Collection, Manassas, VA, USA) were grown, according to [2], in DMEM/F12 medium (Gibco-Invitrogen, Cergy-Pontoise France) containing 10% heat-inactivated fetal calf serum (56 °C for 30 min), antibiotics (100 IU/mL penicillin, 100 µg/mL streptomycin) (Gibco), and 1 mM sodium pyruvate (Gibco). The cells were seeded at 25–32 × 10^3/cm^2 in 75-cm^2 tissue cultured flasks (Falcon) containing 13 mL of culture medium. They were incubated at 37 °C in a humidified atmosphere of air containing 5% CO_2. The culture medium was changed every 2 days, and the cells were passaged once a week by trypsinization (0.05% trypsin-0.02% EDTA) (Gibco).

ARPE-19 cells were exposed to 20 µg/mL of LPS (0128:B12) and co-treated during 24 h with *trans*-resveratrol at 50, 30, 10 or 1 µM (see above for origin and purity) according to [3]. Cell supernatant were collected and the levels of 6 cytokines (IL-8, IL-1b, IL-6, IL-10, TNF α, and IL-12p70) were measured by flow cytometry using multiplex Cytometric Bead Array Kit (BD Bioscience). Finally, the level of the major angiogenic factor VEGF-A was measured in these media by ELISA (eBioscience, Paris, France).

References

1. Blobaum, A.L.; Marnett, L.J. Structural and functional basis of cyclooxygenase inhibition. *J. Med. Chem.* **2007**, *50*, 1425–144
2. Dugas, B.; Charbonnier, S.; Baarine, M.; Ragot, K.; Delmas, D.; Ménétrier, F.; Lherminier, J.; Malvitte, L.; Khalfaoui, T.; Bron, A.; *et al.* Effects of oxysterols on cell viability, inflammatory cytokines, VEGF, and reactive oxygen species production on human retinal cells: Cytoprotective effects and prevention of VEGF secretion by resveratrol. *Eur. J. Nutr.* **2010**, *49*, 435–446.
3. Latruffe, N., Lançon, A., Frazzi, R., Aires, V., Delmas, D., Michaille, JJ., Djouadi, F., Bastin, J., Cherkaoui-Malki, M. Exploring new ways of regulation by resveratrol involving miRNAs, with emphasis on inflammation. *Ann. N. Y. Acad. Sci.* **2015**, *1348*, 97–106.
